# *In vivo* gene expression of *Pseudomonas putida* KT2440 in the rhizosphere of different plants

**DOI:** 10.1111/1751-7915.12037

**Published:** 2013-02-25

**Authors:** Matilde Fernández, Susana Conde, Estrella Duque, Juan-Luis Ramos

**Affiliations:** 1 Bio-Iliberis Research and Development, I+D Department18210, Peligros, Granada, Spain; 2 Department of Environmental Protection, Estación Experimental del Zaidín, Consejo Superior de Investigaciones Científicas-EEZ, 18008, Granada, Spain

## Abstract

*Pseudomonas putida* KT2440 has the ability to colonize the rhizosphere of a wide range of plants and can reach cell densities in the range of 10^5^–10^6^ cfu g soil^−1^. Using the IVET technology we investigated which KT2440 genes were expressed in the rhizosphere of four different plants: pine, cypress, evergreen oak and rosemary. We identified 39 different transcriptional fusions containing the promoters of annotated genes that were preferentially expressed in the rhizosphere. Six of them were expressed in the rhizosphere of all the plant types tested, 11 were expressed in more than one plant and the remaining 22 fusions were found to be expressed in only one type of plant. Another 40 fusions were found to correspond to likely promoters that encode antisense RNAs of unknown function, some of which were isolated as fusions from the bacteria recovered in the rhizosphere from all of the plants, while others were specific to one or several of the plants. The results obtained in this study suggest that plant-specific signals are sensed by KT2440 in the rhizosphere and that the signals and consequent gene expression are related to the bacteria's successful establishment in this niche.

## Introduction

The rhizosphere is a complex ecosystem where a number of dynamic interactions take place between the plant root that exudes a variety of organic molecules and inorganic ions, and prokaryotic and eukaryotic soil microbes and invertebrates. These interactions are also influenced by the physicochemical conditions of the soil. This set of reciprocal interactions causes a network of genetic and physiological responses at the single cell level that have not been fully addressed (Ramos-González *et al*., 2005; Hartmann *et al*., 2009). Analyses of bacterial diversity in the rhizosphere using culture-dependent and culture-independent techniques have yielded large amounts of data relating to the microbes that inhabit this niche and their population levels. Bacteria of the genus *Pseudomonas* are one of the prominent genera of root-associated bacteria, and have been found to be ubiquitous in the rhizosphere of wild and cultivated plants (Haas and Défago, 2005; Uroz *et al*., 2007; Hartmann *et al*., 2009; García-Salamanca *et al*., 2013); however, the responses of these microorganisms to root compounds have not been explored in great detail (Costa *et al*., 2007; Hartmann *et al*., 2009).

*Pseudomonas putida* KT2440 is an efficient root colonizer of a wide range of plants (Molina *et al*., 2000) and is frequently used as a model organism in studies focusing on rhizoremediation, the removal of pollutants by a microbe when associated with the root of plants (Segura *et al*., 2009). Evidence for the reciprocal interactions, between the bacterium and the plant roots, comes from the knowledge that this strain exhibits increased catabolic activity in rhizosphere soil when compared with bulk soil (Fernández *et al*., 2012a). Colonization of roots by KT2440 has been studied in detail using the wild-type strain and a large collection of mutants which had lost their ability to adhere to seeds (Espinosa-Urgel *et al*., 2000; 2002; Duque *et al*., 2013), *in vivo* induced gene expression using IVET technology (Ramos-González *et al*., 2005) and transcriptional profiling of KT2440 proliferating on the roots of plants grown on vermiculite has been addressed (Matilla *et al*., 2007).

IVET is essentially a promoter-trapping technique that selects microbial promoters that are active in a specific habitat. These promoters are selected for their ability to drive the expression of a promoterless selection gene marker *in vivo*, which complements a mutation in an essential gene in the host strain and, therefore enables survival in the tested environment. In contrast to classical approaches by mutagenesis, the advantage of the IVET strategy is the positive selection of genes that are specifically induced by environmental factors (Rediers *et al*., 2003). The specific IVET system used previously by our group was taken for the identification of *P. putida* KT2440 in the rhizosphere of maize plants (Ramos-González *et al*., 2005). In the case of IVET promoters selected in the rhizosphere these promoters are of interest to drive heterologous gene expression such as catabolic genes to remove pollutants or promoters that drive expression of genes involved in biocontrol. The IVET system utilized consists of a plasmid, named pOR1, containing a promoterless ′*asd-lacZ* reporter cassette, which is used to generate a KT2440 promoter library with DNA fragments in the range of 1000 bp, and a suitable host to select the active transcriptional fusions, *P. putida* KT2440Δ*asd* which is an auxotroph that requires diaminopimelate (DAP) and three amino acids: lysine, methionine and threonine for growth. The viability of *P. putida* KT2440Δ*asd* to grow in the rhizosphere is impaired unless the *asd* gene is expressed *in trans* from an active promoter cloned in pOR1. In culture medium, in addition to the auxotrophy, activity of the promoters cloned into pOR1 can be measured using the blue/white screening technique on plates with X-gal.

In this study we used the IVET system designed by Ramos-González and colleagues (2005) to perform a comparative study of *P. putida* KT2440 gene expression in the rhizosphere of four different but representative Mediterranean plants, *Pinus halepensis* (pine), *Quercus ilex* (evergreen oak), *Cupressus sempervirens* (cypress) and *Rosemarinus officinalis* (rosemary). The aim was to determine which genes were preferentially activated when the strain colonized the rhizosphere and to gain insight into how this bacterium is capable of colonizing the roots of different plants.

## Results and discussion

### *Pseudomonas putida* KT2440 has a similar way of colonizing the rhizosphere of different types of plants

**Figure 1:**
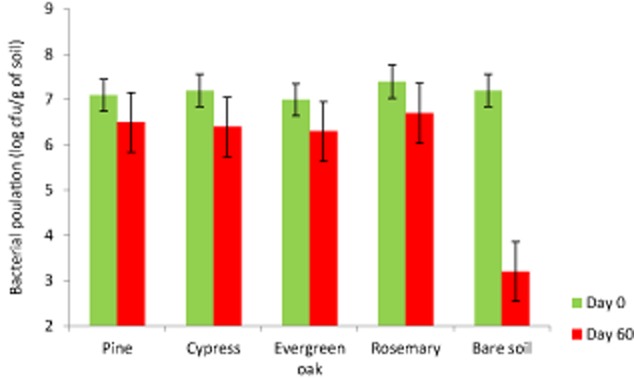
Cell density of *P. putida* KT2440R populations in the rhizosphere of different plants. Grey bars: population sizes the day of inoculation; dark bars: population size 2 months later. The plant species used are indicated. To facilitate the strain identification by plate counting we used KT2440R, a KT2440 rifampicin-resistant spontaneous mutant. In addition, colonies were confirmed by REPc profile analysis (Aranda-Olmedo *et al*., 2002). Rhizosphere colonization assays were performed with young trees in a cambisol soil according to our previously published standard protocol (Fernández *et al*., 2012a). Assays were run in triplicate. Error bars show the standard error.

We assayed the ability of KT2440 to colonize some of the more common plants on the Mediterranean basin: pine, evergreen oak, cypress and rosemary. With this aim we used seedling trees which were inoculated by dipping the root in a bacterial suspension of KT2440, then the plants were placed in pots with a non-sterilized mixture of peat:sand (1:1) and incubated under controlled growth conditions in a greenhouse (23°C, 12 h:12 h light:dark). In the rhizosphere of the four plants, bacteria survived and bacterial density was between 10^5^ and 10^6^ cfu per gram of rhizosphere soil over a period of more than two months (Fig. 1); these data are in agreement with that reported by Molina and colleagues (2000) of KT2440 colonizing the rhizosphere of corn, broad beans and other herbaceous plants. In the bulk soil of the pots, KT2440 bacterial levels dropped to 10^3^ cfu per gram of soil, which was also in agreement with previous observations (Molina *et al*., 2000).

### Unravelling genes/promoters uniquely expressed in the rhizosphere by IVET technology

**Figure 2:**
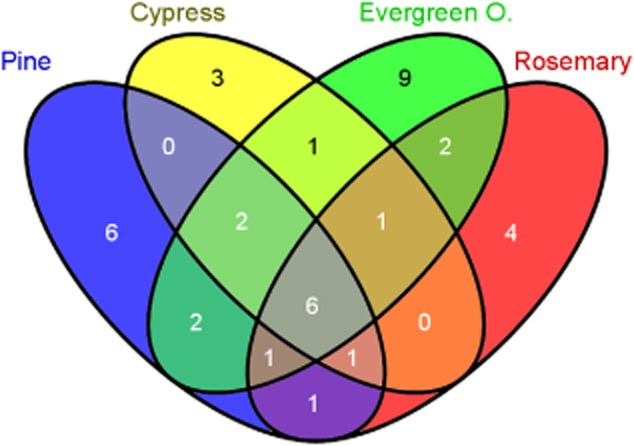
Venn diagram depicting the distribution among the tested plants of the 39 genes identified as preferentially expressed in the rhizosphere during this study. Diagram produced by Venny (Oliveros, 2007).

In this study we aimed to document which genes are specifically expressed in the rhizosphere of the four plants: *P. putida* Δ*asd* bearing the IVET library was inoculated as above and the plants were placed in pots with peat:sand (1:1) as substrate. We performed two independent rounds of IVET selection, with each plant tested in triplicate on each round. All plants were kept for 2 months under greenhouse conditions before recovering the viable bacterial cells on selective medium. Near 50 000 isolated after spreading serial dilutions in minimal medium agar plates with X-gal, kanamycin, DAP, lysine, methionine and threonine. Around 800 of them (1.6% of the total) formed white or very pale-blue colonies; and were unable to grow in the absence of DAP. This was indicative that the promoter that drove *asd* expression was expressed in the soil and not in the agar plates. For each clone the pOR1 derivative was recovered (Rainey *et al*., 1997) and the insert sequenced. Computational analysis of the sequences allowed us to identify a total of 79 different insertions; 39 of which were adjacent to annotated genes (Table 1), whereas the remaining 40 were antisense transcriptional clones (Table S1). From the group of 39 transcriptional fusions, six of them were isolated from the rhizosphere of all of the plants tested, while 11 were isolated from more than one type of plant, and the remaining 22 transcriptional fusions were recovered from the rhizosphere of only one of the four plants tested; specifically, three of them only in cypress, nine in evergreen oak, six in pine and four in rosemary (Fig. 2).

**Table 1:** Genes identified as preferentially activated in the rhizosphere by IVET screening

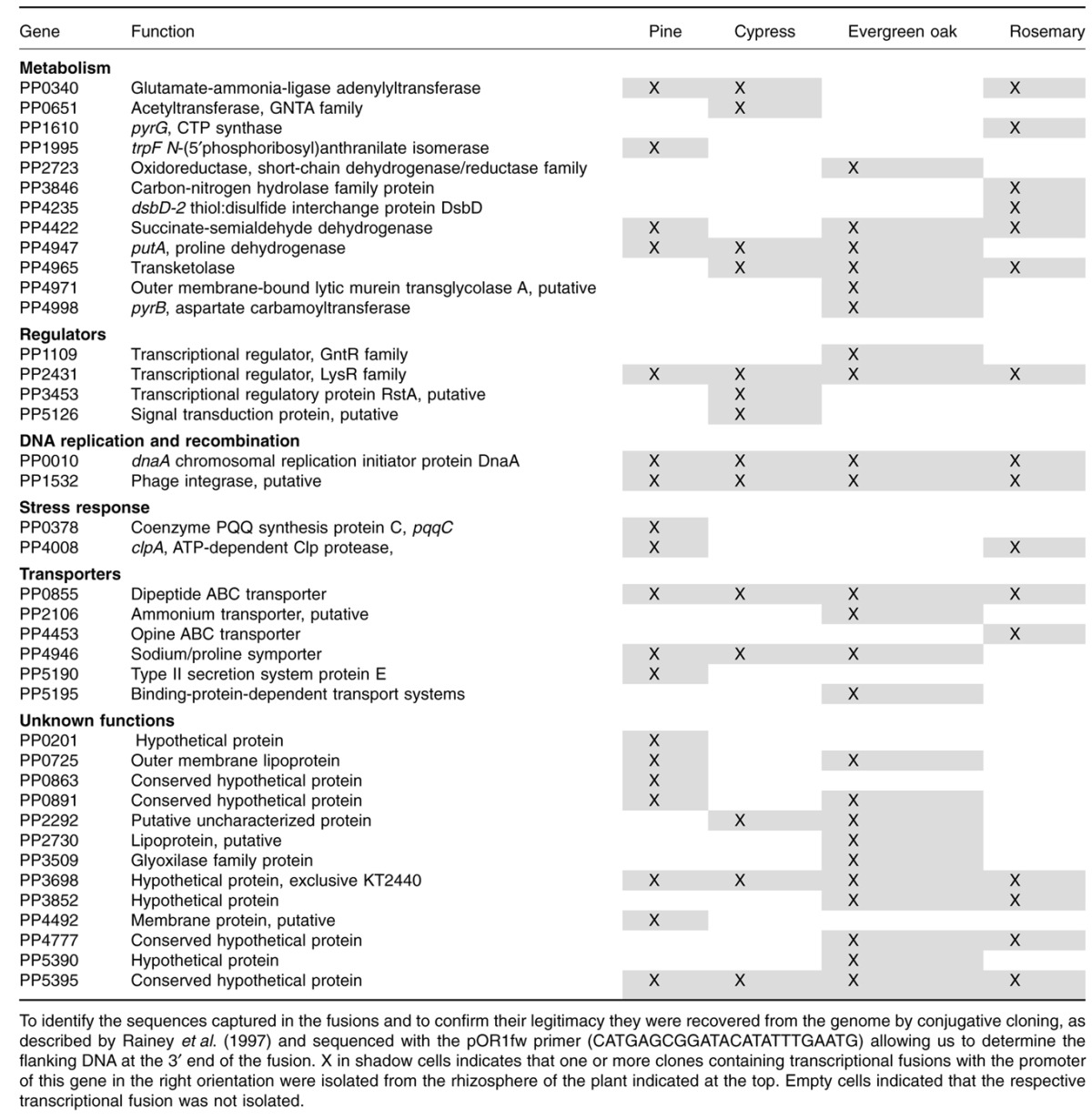

**Figure 3:**
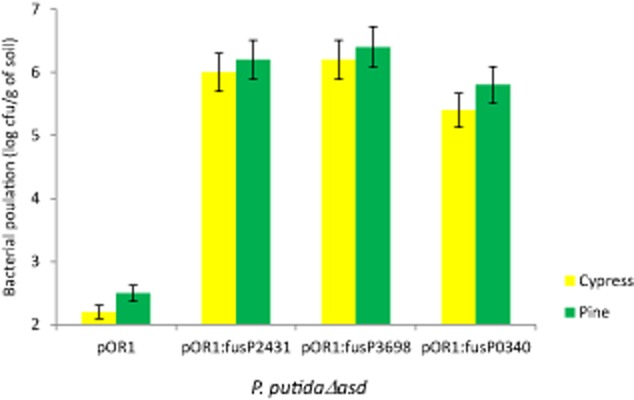
Population size after 2 months in the rhizosphere of *P. putida* △*asd* with pOR1 and different transcriptional fusions in pine and cypress. pOR1: plasmid without insert (negative control); pOR1:fusP2431, transcriptional fusion of the *asd* to the PP2431 promoter, pOR1:fusP3689, transcriptional fusion of the *asd* to PP3689 promoter, and pOR1:fusP0340, transcriptional fusion the *asd* with the PP0340 promoter. Population sizes were determined by plate counting in minimal medium supplemented with kanamycin, DAP and amino acids. Experiments were run in triplicate. Transcriptional fusions to verify were chosen at random, error bars show the standard error.

Although most of the transcriptional fusions were isolated several times from independent samples, we performed an additional set of assays to verify that the isolated clones indeed exhibited *asd* expression in the rhizosphere from the promoters cloned in the transcriptional fusions. With this aim we randomly chose clones containing a transcriptional fusion found in all plants or only in some of the plants and carried out rhizosphere colonization assays with pure cultures of these clones. The results were compared with those obtained with *P. putida* Δ*asd* carrying pOR1 (without insert). In all cases, clones with transcriptional fusions kept their population sizes at a level that was at least three orders of magnitude higher than *P. putida* Δ*asd* with the empty plasmid (Fig. 3). When clones with transcriptional fusions found in only one type of plant were tested in the rhizosphere of another plant, differences in the cell densities were significant, around two orders of magnitude of variation (Table 2).

**Table 2:** *Pseudomonas putida* KT2440 population size after 2 months in the rhizosphere of different plants

	cfu per gram of rhizosphere soil		
	pOR1	pOR1:fusPP4492	pOR1:fusPP1109
Pine	3.6 × 10^2^ ± 8	7.9 × 10^5^ ± 27	3.9 × 10^4^ ± 57
Evergreen oak	1.8 × 10^2^ ± 10	3.1 × 10^3^ ± 61	2.5 × 10^6^ ± 94

pOR1: plasmid without insert (negative control); pOR1:fus4492, transcriptional fusion of the *asd* to the PP4492 promoter; pOR1:fus1109, transcriptional fusion of the *asd* to the PP1109 promoter. Bacterial densities were determined by plate counting in minimal medium supplemented with kanamycin, DAP and amino acids. Experiments were run in triplicate. Transcriptional fusions to verify were chosen at random.

### Specificity of the rhizosphere-activated genes

**Figure 4:**
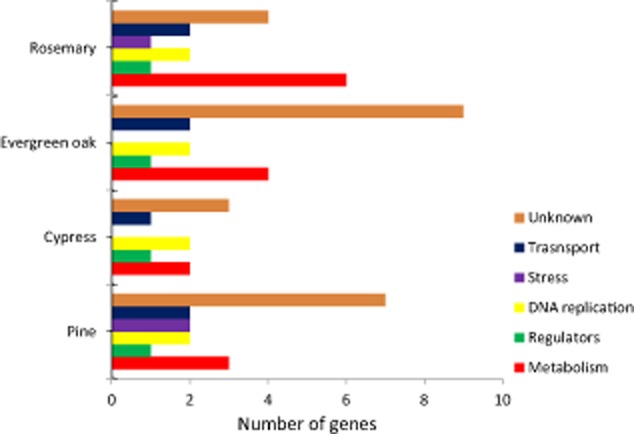
Functional classification of the genes identified as preferentially activated in the rhizosphere by the IVET screening technology. Functional categories have been assigned according to *in silico* predictions. Only genes whose promoters were trapped in the right sense have been included.

To further investigate the putative role of the activated genes in rhizosphere adaptation *in silico* studies were carried out (see below), as well as a detailed analysis of the previously published information on the function of these genes. Functional analysis of the genes expressed from the promoters contained in the transcriptional fusions, revealed six main categories: metabolism, gene regulation, DNA replication and recombination, stress response, transporters, and a number of hypothetical proteins of unknown function (Fig. 4). This reinforces the hypothesis that a strict signalling and a complex network of interactions occur in the rhizosphere enabling bacteria to cope with two general problems: new nutrient sources and significant stress conditions (Hartmann *et al*., 2009). These categories are in agreement with those described for KT2440 in the rhizosphere of maize grown in hydroponic solution (Ramos-González *et al*., 2005) and sand (Matilla *et al*., 2007), although the genes identified in the current study are different.

### Transcriptional fusions found in the rhizosphere of all or several types of plants

As detailed above, six transcriptional fusions were expressed in the rhizosphere of all of the plants tested. This group includes the promoters of PP0885, encoding a dipeptide ABC transporter which was shown to be upregulated in KT2440 under nitrogen limitation conditions (Hervás *et al*., 2008). Also induced is the phage integrase encoded by PP1532 that Quesada and colleagues (2012) found to belong to a prophage whose mobilization, mediated by the expression of PP1532, resulted in increased bacterial fitness in the rhizosphere; and that is also activated in response to quorum sensing signals induced by a *Pseudomonas aeruginosa* strain (Fernández-Piñar *et al*., 2011). Significant as well is the expression in all of the plants of a transcriptional regulator encoded by PP2431 which can be relevant for cellular adaptation at the genetic level. Also a fusion with *dnaA* promoter was solely activated in the rhizosphere; this gene is involved in DNA replication, initiation and regulation. Finally, two of the transcriptional fusions found in the four types the plants tested (PP5395 and PP3698) corresponded to genes of unknown function that are exclusively found in the KT2440 strain.

A set of 11 transcriptional fusions were isolated from more than one type of plant; among these transcriptional fusions were genes involved in stress tolerance, such as *clpA* or PP4422, involved in phenol tolerance in KT2440 (Putrins *et al*., 2010); genes with a metabolic function, i.e. *glnE* (PP4965) encodes a transketolase, which was also induced in *Streptomyces coelicolor* in the presence of plant material (Langlois *et al*., 2003). In three of the plants we found transcriptional fusions with two genes involved in proline transport and metabolism; the sodium/proline symporter encoded by PP4946, which was also identified as being activated in the rhizosphere of corn (Ramos-González *et al*., 2005) and *putA*, involved in proline catabolism (Vílchez *et al*., 2000). It was previously shown that *putA* expression is induced in KT2440 in response to maize root exudates (Vílchez *et al*., 2000), and more recently it has been identified to be induced by a benzoxazinoid, a secondary metabolite involved in plant defence (Neal *et al*., 2012). Signals for the activation of this group of common genes could be non-specific plant-derived factors or the surrounding microbial signals, without ruling out a combination of both of them together with abiotic elements (Haas and Défago, 2005).

### Transcriptional fusions found in only one type of plant

Up to 22 transcriptional fusions were expressed in the rhizosphere of only one of the four tested plants; six of them only in pine, three of them in cypress, nine in evergreen oak and four in rosemary; this group also includes a set of fusions with promoters of genes involved in metabolic processes, transporters, regulators and bacterial stress responses.

Exclusively in the rhizosphere of pine we detected the activation of *trpF* (PP1995), involved in tryptophan biosynthesis, which in turn is the precursor for indole-3-acetic acid (IAA), a well-characterized phytohormone able to stimulate plant growth and several responses in plants (Cleland, 1990; Hagen, 1990; Patten and Glick, 1996; Spaepen *et al*., 2007; Roca *et al*., 2012). A second gene of this group is *pqqC* (PP0378), involved in the biosynthesis of pyrroloquinoline quinone coenzyme (PQQ), which in *Pseudomonas* is involved in the stress response (Fernández *et al*., 2012b), phosphate solubilization (Meyer *et al*., 2011) and have direct effects on plant growth (Choi *et al*., 2008).

Two transcriptional fusions with promoters of genes involved in bacterial signalling/regulation processes, PP3453 and PP5126, were isolated only from the cypress rhizosphere. *In silico* searches using Search Tool for the Retrieval of Interacting Genes/Proteins (STRING 9.0) database (http://string-db.org/) predicted the interaction of the signal transduction protein encoded by PP5126 with four different flagellar motor proteins.

One transcriptional fusion was to the orf PP5390 and it was found in bacteria grown in the evergreen oak rhizosphere, this gene has been previously described as induced by maize root exudates (Ramos-González *et al*., 2005), and encodes a protein of unknown function.

Among the transcriptionl fusions isolated only in the rhizosphere of rosemary we found the promoter region of PP4453, encoding an opine ABC transporter; since KT2440 carries the set of genes that encodes enzymes for the metabolism of opines, we suggest that induction of this transporter is related to the ability of this bacterium to grow at the expense of plant-produced opines (Nelson *et al*., 2002).

### Antisense transcriptional fusions

Half of the transcriptional fusions isolated in this study lacked a detectable promoter or the captured DNA was oriented in the opposite direction to that necessary for transcription of the promoterless *asd* gene. Even accepting that some of them could be false positives, the number of such clones and the fact that most of them were recovered on independent occasions and were isolated from some of the tested plants or in all of them, strongly suggested an active role of the fragment trapped in such fusions. Antisense transcriptional fusions are often found in studies using IVET approaches (Silby *et al*., 2004; Ramos-González *et al*., 2005; Barr *et al*., 2008; Silby *et al*., 2009; Hanin *et al*., 2010). Authors have speculated on their role as antisense regulatory RNAs; Frank and colleagues (2011) demonstrated the existence of non-coding RNAs in KT2440 in lab conditions, which probably also take place *in planta*. Also it has been considered that putative errors in *in silico* gene prediction or even the existence of overlapping protein-coding genes that run in opposite direction to the annotated ORFs (Silby *et al*., 2004). In any case, there is much to learn about the role of these genes in the bacterial natural environments (Silby *et al*., 2009). Therefore it is quite possible that we may have detected only a selection of a vast genetic response allowing this bacterium to colonize the rhizosphere of very different types of plants, and these cryptic fusions suggest the existence of genes whose expression in the rhizosphere seems to be independent of the type of plant, and genes whose expression strongly depends on the type of plant.

Root exudates are considered the main factor for changes in bacterial gene expression in the rhizosphere. These exudates are plant-specific and can also vary depending on the plant growth cycle or on the physiological state (Jones *et al*., 2004; van Veen *et al*., 2007; Hartmann *et al*., 2009). Differences in root exudate composition between different plants have also been suggested as the main reason for the changes observed in bacterial rhizospheric communities depending on the type of plant (Hartmann *et al*., 2009). Mark and colleagues (2005) analysed how the exudates of two varieties of sugar beet influenced the pattern of genetic expression of *P. aeruginosa*. They found that each exudate provoked a different transcriptional response with only a partial overlap. Such influences of the root exudate composition on bacterial gene expression or on microbial populations contrasts with the fact that while some microbes exhibited a very narrow spectrum of plant roots to colonize, there are others, considered almost ubiquitous in the rhizosphere. Results from this study fully support the influence of plant-specific factors, most probably root exudates, on microbial adaptation to the rhizosphere environment, but possibly in combination with other (still) unknown factors.

In summary, *P. putida* KT2440 efficiently colonizes the rhizosphere of a wide range of plants and apart from the plasticity of the genome of this strain; successful colonization involves a response to plant-specific signals, which is undoubtedly part of its success in this specific ecological niche.

## Supporting Information

Table S1 Cryptic transcriptional fusions.
