# Supplementary material for: In vivo gene expression of Pseudomonas putida KT2440 in the rhizosphere of different plants
Source: Microb Biotechnol. 2013 Feb 25;6(3):307–13. doi: 10.1111/1751-7915.12037 (PMC3815925; doi:10.1111/1751-7915.12037)
Supplement: Table S1 — Cryptic transcriptional fusions. [file mbt0006-0307-sd1.docx]

| **GENE ON OPPOSITE STRAND** | | **PLANT** | | | |
| --- | --- | --- | --- | --- | --- |
|  | |  | | | |
| Gene | Function | Pine | Cypress | Evergreen Oak | Rosemary |
|  |  |  |  |  |  |
| PP0013 | Hypothetical | X |  |  |  |
| PP0121 | Homoserine kinase | X | X |  | X |
| PP0204 | Transcriptional regulator, GntR family | X |  |  |  |
| PP0288 | Oxalate/Formate antiporter | X | X | X | X |
| PP0651 | Acetyltransferase, GNTA family |  | X |  |  |
| PP0660 | Aminoacid transporter | X |  |  |  |
| PP0678 | Conserved hypothetical protein |  |  |  | X |
| PP0725 | TPR domain protein | X |  |  |  |
| PP0899 | Ferredoxin Reductase | X | X | X | X |
| PP1445 | Porin B |  | X |  | X |
| PP1488 | Methyl-accepting chemotaxis transducer |  |  |  | X |
| PP1588 | Aminotransferase, class I |  | X |  |  |
| PP1593 | Uridylate kinase, ribosome recycling factor | X | X | X |  |
| PP1627 | Conserved hypothetical protein |  | X |  |  |
| PP1791 | Aldolase/synthase |  |  |  | X |
| PP1889 | Type 1 pili subunit FimD | X | X |  | X |
| PP2684 | Hypothetical protein |  |  |  | X |
| PP2911 | Gamma-aminobutyrate transporter |  |  | X |  |
| PP2977 | Transposase | X | X | X | X |
| PP3124 | Short-chain fatty acid transporter |  |  | X | X |
| PP3127 | Exopolysaccharide transport protein, putative |  |  | X |  |
| PP3167 | benzoate transport protein | X |  |  |  |
| PP3689 | Serine/threonine protein phosphatase |  |  | X | X |
| PP3694 | conserved hypothetical protein ( | X |  |  |  |
| PP3772 | Repressor related protein |  |  |  | X |
| PP4021 | Esterase |  |  | X |  |
| PP4042 | Glucose 6-phosphate 1-dehydrogenase |  |  |  | X |
| PP4047 | hypothetical protein | X |  |  | X |
| PP4115 | conserved hypothetical protein |  | X |  |  |
| PP4169 | Glycerol-3-phosphate dehydrogenase |  |  | X | X |
| PP4301 | pyruvate kinase I |  | X |  |  |
| PP4304 | cation transporter, VIC family |  | X | X | X |
| PP4363 | sensor histidine kinase/response regulator | X |  | X |  |
| PP4449 | hypothetical protein | | X |  | X |
| PP4703 | conserved hypothetical protein |  |  | X |  |
| PP4739 Putative protein | | X | X |  | X |
| PP4772 | ATP-dependent helicase HrpB | X | X | X |  |
| PP4796 | DNA polymerase III, delta subunit | X |  |  | X |
| PP5080 | type IV pili biogenesis protein PilQ | X |  |  |  |
| PP5214 | transcription termination factor Rho |  | X | X |  |

**Supplemental Table 1.- Cryptic transcriptional fusions**. Genes whose promoters were found trapped in the opposite sense to those necessary for their transcription or those where an internal gene fragment was cloned. X in shadow cells indicates that one or more clones containing the corresponded transcriptional fusions were isolated on the rhizosphere of the plant indicates in the top. Empty cells indicated that the respective transcriptional fusion was not isolated
